# 

**Published:** 1890-01-04

**Authors:** 


PRICE ONE PENNY.

No. 171, Vol. VII.—January 4th, 1890.

[Registered at the General Post Office as a Newspaper for transmission in the United Kingdom and Abroad.

WITH EXTRA NURSING SUPPLEMENT.
Per Annum, 6s. 6d., post free.
Monthly Parts, 6d.; post free 7½d.
Ditto per Annum, 7s. 6d., post free.

### THE HOSPITAL

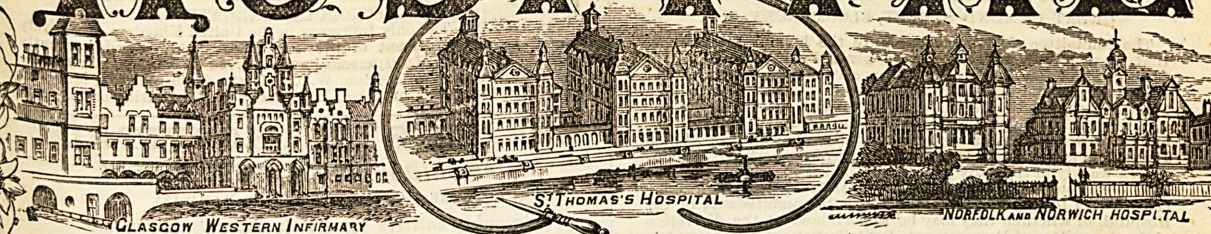

A WEEKLY JOURNAL OF

Science, Medicine, Nursing & Philanthropy.

SATURDAY, JANUARY 4th, 1890.

A Promise of Dawn - 209
Words of Consolation—A Happy New Year - 210
January - 211
The History and Capabilities of Herbal Simples.—II.—A Eulogy, and Some Ancient Authorities. By W. T. Fernie, M.D. - 212
The Doctor's Pulpit.—"Confusion Worse Confounded" - 213
Notes and News - 214
Everybody's Page - 216
Annotations.—Influenza, Scarlet Fever, and Dengue—The Drawing Room and the Epidemic - 217
The Philanthropist's Vade Mecum.—General Hospitals, Hospitals for Consumption, Hospitals for Children, 219; Hospitals for Women, Lying-In Hospitals, Nervous Diseases, Epilepsy and Paralysis, 220; Temperance and Cancer, Hospitals for Incurables, Miscellaneous Special Hospitals, Dispensaries, Nursing Institutions, 221; A Few Other Charities - 222

EXTRA SUPPLEMENT—THE NURSING MIRROR.

En Passant.—Doll Nurses—Church Army Nurses—Nurses for India—Northampton Institute—A Nurse's Case-Book—Short Items—A Letter from the States—Grumbling Letters - lvii
Notes on Opiates - lviii
In Days of Sickness - lviii
Everybody's Opinion.—Monthly Nurse and Midwife Registration - lix
Keeping Christmas - lx
Presentations - lx
Death in Our Ranks - lx
Vacancies—Off Duty - lx

